# Lack of mutation at codon 531 of *SRC* in advanced colorectal cancers from Italian patients

**DOI:** 10.1054/bjoc.2000.1560

**Published:** 2001-01

**Authors:** L Laghi, P Bianchi, O Orbetegli, L Gennari, M Roncalli, A Malesci

**Affiliations:** Departments of Gastroenterology, Pathology, Surgery, Research Laboratory, Istituto Clinico Humanitas, Via Manzoni 56, Rozzano, Milan, 20089, Italy; Departments of Pathology, Internal Medicine, University of Milan, Via Festa del Perdono 7, Milan, 20122, Italy

**Keywords:** colorectal cancer, proto-oncogenes, c-Src tyrosine kinase, Italy

## Abstract

A truncating mutation (C to T transition) at codon 531 of the human protooncogene c-src, possibly accounting for the activation of c-src tyrosine kinase, has been recently identified in a subset of advanced colorectal cancer from North-American patients. However, two subsequent studies have failed to confirm the occurrence of *SRC* 531 mutation in colorectal cancers from North-European and Asiatic patients, raising the hypothesis that the genetic activation of src in colon cancer might be restricted to patients belonging to specific ethnic groups. We investigated a large series of colorectal cancers from Italian patients (155 cases) with a high prevalence of liver metastasis (43%). Using a PCR-RFLP assay, the occurrence of a *SRC* 531 mutation was ruled out in all the investigated specimens of primary tumours and/or metastases. Our results demonstrate that *SRC* Gln531AMB plays no role in the development or in the progression of colorectal cancer among Italian patients. © 2001 Cancer Research Campaign http://www.bjcancer.com


British Journal of Cancer (2001) 84(2), 196–198
© 2001 Cancer Research Campaign
doi: 10.1054/ bjoc.2000.1560, available online at http://www.idealibrary.com on IDEAL®

http://www.bjcancer.com

Short Communication

### Lack of mutation at codon 531 of *SRC* in advanced colorectal cancers from Italian patients

**L Laghi[1,4], P Bianchi[2,4], O Orbetegli[1,4], L Gennari[3], M Roncalli[2,5] and A Malesci[1,6]**

Departments of [1]Gastroenterology, [2]Pathology, [3]Surgery and [4]Research Laboratory, Istituto Clinico Humanitas, Via Manzoni 56, Rozzano, Milan, 20089 Italy; Departments of [5]Pathology and [6]Internal Medicine, University of Milan, Via Festa del Perdono 7, Milan 20122, Italy

**Summary** A truncating mutation (C to T transition) at codon 531 of the human protooncogene c-src, possibly accounting for the activation of c-src tyrosine kinase, has been recently identified in a subset of advanced colorectal cancer from North-American patients. However, two subsequent studies have failed to confirm the occurrence of *SRC* 531 mutation in colorectal cancers from North-European and Asiatic patients, raising the hypothesis that the genetic activation of src in colon cancer might be restricted to patients belonging to specific ethnic groups. We investigated a large series of colorectal cancers from Italian patients (155 cases) with a high prevalence of liver metastasis (43%). Using a PCR-RFLP assay, the occurrence of a *SRC* 531 mutation was ruled out in all the investigated specimens of primary tumours and/or metastases. Our results demonstrate that *SRC* Gln531AMB plays no role in the development or in the progression of colorectal cancer among Italian patients. © 2001 Cancer Research Campaign http://www.bjcancer.com

**Keywords**: colorectal cancer; proto-oncogenes; c-Src tyrosine kinase; Italy

Elevated levels of c-Src tyrosine kinase activity have been documented in several types of human primary cancers, and particularly in colorectal cancer (Ottenhoff-Kalff et al, 1992; Talamonti et al, 1993). Thus, activated expression of c-Src, a cellular human proto-oncogene homologous to the *v-src* gene of Rous sarcoma virus, has been postulated to play an important role in the development or progression of human colon cancer (Mao et al, 1997). Recently, a novel activating mutation of *SRC* (C to T transition at codon 531) has been described in 12% of advanced human colorectal cancers from North America, raising the possibility of a mutational activation of src kinase in these tumours (Irby et al, 1999). The *SRC* 531 mutation has been then characterized as activating, transforming, tumorigenic and metastasis-promoting in experimental models. However, two subsequent studies failed to detect any *SRC* 531 mutation in colorectal cancers from Japanese and North European (Daigo et al, 1999), as well as from Chinese (Wang et al, 2000) patients. Differences in ethnicity or in the prevalence of advanced cancer between the individual series might account for the observed discrepancy.

The aim of our study was to look for *SRC* 531 mutations in a large series of colorectal cancers from Italian patients, with a high prevalence of metastatic tumours.

### MATERIALS AND METHODS

### Tissue specimens and DNA extraction

To see at which time *SRC* 531 mutations could possibly occur along the progression of colorectal cancer, we examined 119 tissue specimens of primary colon cancer at different stages, and 52 samples of synchronous or metachronous liver metastases, for a total of 155 cases (Table 1). All tissues were obtained from Italian patients who had undergone surgical resection of the primary tumour or of metastases at the Istituto Clinico Humanitas between January 1997 and June 1999. Corresponding normal tissue samples were always available.

*Received 3 May 2000*
*Revised 12 September 2000*
*Accepted 18 September 2000*

*Correspondence to:* A Malesci[1]. E-mail: alberto.malesci@humanitas.it

DNA extraction from paraffin-embedded tumour specimens was carried out by digestion with Proteinase K (100 μg $ml^{-1}$) at 37˚C for 12 h, followed by proteinase heat inactivation.

### PCR amplification and RFLP analysis

A 149 bp amplicon of *SRC* exon 12 (GenBank accession K03218) was obtained using the following primers: 5'-AGTGCTGGCG-GAAGGAGCCT-3' (forward) and 5'-ATCCAAGCCGAGAAGC-CGGT-3' (backward). The wild-type allele was amplified from normal placental human DNA (human COT, Gibco Life Technologies, Paisley, UK). PCR was run for 35 cycles, each cycle consisting of denaturation at 94˚C for 30 s, annealing at 55˚C for 30 s and extension at 72˚C for 30 s.

A *Sca*I-based RFLP-PCR assay was employed to detect the C to T transition at the first base of codon 531 leading to AMBER mutation. In the assay, only amplicons with the mutated TAG sequence are cut by *Sca*I, their digestion resulting in two fragments of 90 and 59 bp, respectively. A positive control was PCR-generated using a 58-bp megaprimer containing the C to T transition at the first base of codon 531 of *SRC* (from nucleotide 254 to nucleotide 197 of K03218, with A instead of G at position 198).

Digestion of amplicons was tested after overnight incubation with 5 U of *Sca*I (New England Biolabs, Beverly, MA, USA). The efficiency of the *Sca*I digestion in detecting variable amounts of SRC531 mutated DNA was tested by assaying scalar ratios of mutated to wild-type allele.

### RESULTS

The *Sca*I RFLP assay detected the mutated allele when the ratio between mutated and wild-type PCR products was 1:8 or higher (Figure 1).

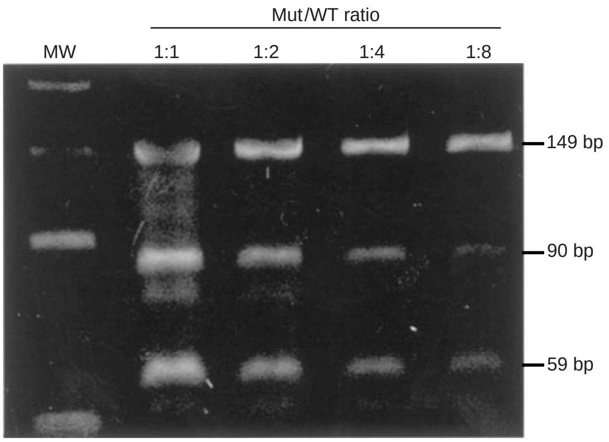

**Figure 1** *Sca*I RFLP of codon 531-mutated *SRC* exon 12, in a scalar ratio to wild-type. 90 and 59 bp digestion products are detectable up to a 1:8 ratio of mutated-to-wild-type allele

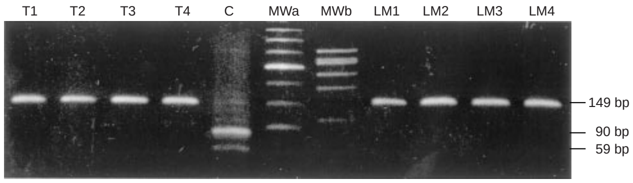

**Figure 2** Agarose gel of RFLP-PCR products from advanced colorectal cancers (T), and from liver metastases (LM). Digested fragments, evident in the PCR-generated positive control (C), are not detectable in any tested sample. MW = molecular weight markers: a = 50 bp ladder; b = *øX174/Hind* III

The expected amplicon was obtained from all the 171 cancer tissues included in the study (Table 1). The RFLP-PCR-assay detected no *SRC* 531 mutation in any of the investigated tissues. A non-digested product of 149 bp was always seen in gels from normal tissues, primary colon cancers, and liver metastases. Conversely, in each gel, the PCR-generated positive control for enzyme digestion showed digested fragments of 90 and 59 bp, respectively (Figure 2).

Direct sequencing of several tumour samples confirmed the presence of a wild-type codon 531, as opposed to the AMBER mutation present in the PCR-generated positive control (Figure 3).

### DISCUSSION

By examining a total of 67 cases of metastatic colorectal cancer (42 Dukes D at time of diagnosis and 25 metachronous liver metastases, Table 1) for the presence of mutation in the primary tumour and/or in the metastases, we expected to detect 5–8 cases with *SRC* 531 mutation according to the originally reported prevalence of AMBER mutation in North American patients (Irby et al, 1999). In fact, no *SRC* 531 mutation was detected in any tissue of our series (Figures 2 and 3).

In their original report, Irby et al (1999) found high levels of c-Src protein kinase activity in tissues positive at mutation analysis, but they did not preselect tissues on this basis. Therefore, the absence of mutations in our consecutive series of late-stage colorectal cancers and liver metastases cannot be the

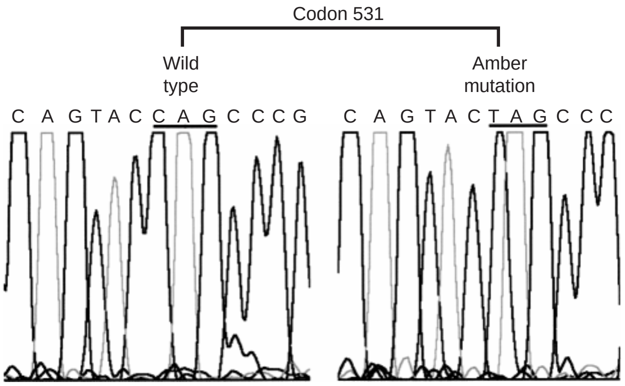

**Figure 3** Direct sequencing of wild-type and mutated *SRC* exon 12 PCR products. At codon 531, the mutation (C to T transition) seen in the megaprimer-generated product, is not present in the PCR product from cancer tissue (wild-type)

result of different criteria of selection. It is also unlikely that our failure in detecting *SRC* 531 mutations may depend on tumour sampling, since Irby et al claimed the genetic change to be clonal in origin and present at multiple sites in each tumour. In addition, the relative percentage of tumour cells in our tissues was always higher than 50%, which, in case of heterozygous mutation, would exceed a 1:4 ratio of mutant to wild-type allele. Thus, negative results at the *Sca*I-based assay can be taken safely (Figure 1), as long as the mutant and the wild-type alleles are assumed to be equally amplified. At any event, a mutant allele-specific test overcoming any preferential amplification of the wild-type allele only confirmed mutations already identified at the RFLP assay (Irby et al, 1999).

As Daigo et al (1999) failed to find any genetic alterations at codon 531 of human *SRC* in a large series of advanced colorectal cancers from the Netherlands and from Japan, the hypothesis was made that the *SRC* 531 mutation might be a mechanism of Src activation in colon carcinogenesis only in a fraction of American patients but not in other ethnically different populations (Daigo et al, 1999). Along this line of investigation, Wang et al (2000) recently reported no *SRC* 531 mutation in a series of colorectal cancers from China. Besides ethnical differences, it must be emphasized that both negative studies investigated series of colorectal cancer with a prevalence of metastases lower than that of the series studied by Irby et al (12% vs 63%). In our study, metastatic colorectal cancers accounted for 43% of the entire series, including 25 metachronous liver metastases. Thus, our negative results conclusively demonstrate that the *SRC* 531 mutation does not confer any growth advantage and does not contribute to clonal expansion or metastasis in colorectal cancer from Italian patients. At this time, the cumulating negative findings in ethnically different populations do question the real

**Table 1** Tumour tissues tested for the *SRC* 531 mutation and initial staging of cancer (number of samples)

| | Dukes stage at diagnosis | | | |
|---|---|---|---|---|
| | A | B | C | D |
| Primary colorectal cancers (*n* = 119) | 4 | 37 | 49 | 29 |
| Liver metastases (*n* = 52) | 1 | 6 | 18[a] | 27[b] |
| | metachronous | | | |

[a]Two matched with primary CRC; [b]14 matched with primary CRC

worldwide occurrence of *SRC* 531 mutation, even in a minor fraction of advanced colon cancer. The possibility that in the original study (Irby et al, 1999) an artificial mutation might have been generated by a technical artifact, should be considered. If it is obvious that such genetic alteration is of no use in identifying patients with potentially metastatic disease, it is also extremely unlikely that the mutation at codon 531 might even partially explain the src activation commonly observed in advanced colorectal cancer. To further investigate a possible c-Src genetic activation in colorectal carcinogenesis, the entire c-Src gene should be investigated for mutations, particularly in tumours with high levels of tyrosine kinase activity.

### REFERENCES

Daigo Y, Furukawa T, Ishiguro H, Fujita M, Sugai S, Nakamori S, Liefers GJ, Tollenaar RAEM, van de Velde CJH and Nakamura Y (1999) Absence of genetic alteration at codon 531 of the human c-src gene in 479 advanced colorectal cancers from Japanese and Caucasian patients. *Cancer Res* **59**: 4222–4224

Irby RB, Mao WN, Coppola D, Kang J, Loubeau JM, Trudeau W, Kael R, Fujita DJ, Jove R and Yeatman TJ (1999) Activating SRC mutation in a subset of advanced human colon cancers. *Nat Genet* **21**: 187–190

Mao W, Irby R, Coppola D, Fu L, Wloch M, Turner J, Yu H, Garcia R, Jove R and Yeatman TJ (1997) Activation of c-Src by receptor tyrosine kinases in human colon cancer cells with high metastatic potential. *Oncogene* **15**: 3083–3090

Ottenhoff-Kalff AE, Rijksen G, van Beurden EA, Hennipman A, Michels AA and Staal GE (1992) Characterization of protein tyrosine kinases from human breast cancer: involvement of the c-src oncogene product. *Cancer Res* **52**: 4773–4778

Talamonti MS, Roh MS, Curley SA and Gallick GE (1993) Increase in activity and level of pp60c-src in progressive stages of human colorectal cancer. *J Clin Invest* **91**: 53–60

Wang NM, Yeh KT, Tsai CH, Chen SJ and Chang JG (2000) No evidence of correlation between mutation at codon 531 of src and the risk of colon cancer in Chinese. *Cancer Lett* **150**: 201–204

 © 2001 Cancer Research Campaign

